# NSD3 in Cancer: Unraveling Methyltransferase-Dependent and Isoform-Specific Functions

**DOI:** 10.3390/ijms25020944

**Published:** 2024-01-12

**Authors:** Yanara Nuñez, Sebastian Vera, Victor Baeza, Valentina Gonzalez-Pecchi

**Affiliations:** 1 Biomedical Science Research Laboratory, Department of Basic Sciences, Faculty of Medicine, Universidad Católica de la Santísima Concepción, Concepción 4090541, Chile; yanara_nunez@hotmail.com (Y.N.); svera@magister.ucsc.cl (S.V.); victor.baeza.ch@gmail.com (V.B.); 2 Biochemistry, Faculty of Pharmacy, Universidad de Concepción, Concepción 4070383, Chile

**Keywords:** cancer, molecular oncology, oncogenes, NSD3, NSD3S

## Abstract

NSD3 (nuclear receptor-binding SET domain protein 3) is a member of the NSD histone methyltransferase family of proteins. In recent years, it has been identified as a potential oncogene in certain types of cancer. The NSD3 gene encodes three isoforms, the long version (NSD3L), a short version (NSD3S) and the WHISTLE isoforms. Importantly, the NSD3S isoform corresponds to the N-terminal region of the full-length protein, lacking the methyltransferase domain. The chromosomal location of NSD3 is frequently amplified across cancer types, such as breast, lung, and colon, among others. Recently, this amplification has been correlated to a chromothripsis event, that could explain the different NSD3 alterations found in cancer. The fusion proteins containing NSD3 have also been reported in leukemia (NSD3-NUP98), and in NUT (nuclear protein of the testis) midline carcinoma (NSD3-NUT). Its role as an oncogene has been described by modulating different cancer pathways through its methyltransferase activity, or the short isoform of the protein, through protein interactions. Specifically, in this review we will focus on the functions that have been characterized as methyltransferase dependent, and those that have been correlated with the expression of the NSD3S isoform. There is evidence that both the NSD3L and NSD3S isoforms are relevant for cancer progression, establishing NSD3 as a therapeutic target. However, further functional studies are needed to differentiate NSD3 oncogenic activity as dependent or independent of the catalytic domain of the protein, as well as the contribution of each isoform and its clinical significance in cancer progression.

## 1. Introduction

Chromatin organization is highly regulated by many factors, one of them being histone post-translational modifications (HPTMs) [1]. These modifications have a direct impact on the level of chromatin compaction and the degree of transcription. Condensed chromatin, or heterochromatin, is associated with transcriptional repression, and loose chromatin, or euchromatin, associated with an active transcription of genes [2]. HPTMs can be recognized by proteins that are referred to as “readers” and are modified by adding or eliminating PTMs by proteins called “writers” or “erasers”, respectively. Histone methyltransferases (HMT) are “readers” and “writers” of the chromatin, responsible for catalyzing the addition of methyl groups in arginine (PRMT) or lysine residues (HKMT) on the N-terminal histone tails. Most histone lysine methylation is carried out by a family of SET-domain containing proteins, which catalyze the addition of one to three methyl groups [3]. The nuclear receptor-binding SET domain (NSD) family of histone lysine methyltransferase is composed of three members: NSD1, NSD2 (MMSET/WHSC1, Wolf-Hirschhorn syndrome candidate 1), and NSD3 (WHSC1L1, WHSC1 like 1). The NSD proteins play a crucial role in regulating chromatin integrity and gene expression by mono- or di- methylating histone H3 lysine 36, generating H3K36me1 and H3K36me2 [4,5]. Alterations in the NSD proteins have been correlated with human diseases. NSD1 haploinsufficiency and point mutations are implicated in Sotos syndrome [6], a childhood developmental disease, prostate cancer, melanoma, and acute myeloid leukemia (AML) [7]. Haploinsufficiency of NSD2 is related to Wolf-Hirschhorn syndrome, multiple myeloma, neuroblastoma, endometrial and hepatocellular cancer, among others [4,8]. Finally, aberrant expression of NSD3 has been implicated in the development of multiple cancer types, such as lung, breast, and pancreatic cancer [9,10,11]. Given the importance that NSD3 has gained in the past few years in cancer development and progression, this article provides a detailed review of NSD3 alterations and involvement of the long and short isoform in important cancer pathways. 

## 2. NSD3 Protein Structure

**Figure 1:**
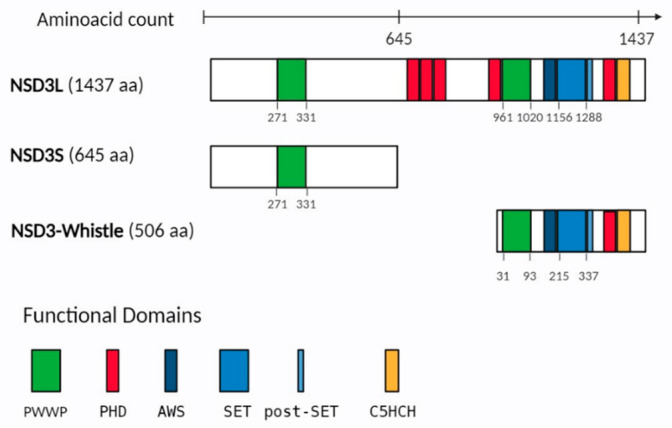
Representation of the functional domains in NSD3 isoforms. NSD3 isoforms, including NSD3L (NSD3-long), NSD3S (NSD3-short) and NSD3 Whistle. Amino acid numbers indicate each functional domain. Different colored rectangles represent the major domains including: PWWP (Pro-Trp-Trp-Pro) in green, PHD (plant homeo domain) in red, AWS (associated with SET), SET and post-SET in blue, and C5HCH (Cys-His-rich domain) in orange. In parentheses, the protein length (amino acid, aa) is mentioned.

NSD3 was first described in 2000 by studying the PWWP (proline-tryptophan-tryptophan-proline) domain of NSD2 and performing a database search for proteins having the PWWP domain in their structure. The NSD3 gene is found on chromosome 8p11.2 [12] and encodes three isoforms by alternative splicing. The long isoform, termed NSD3L, is a protein of 1437 amino acids [13]. Alternative splicing of exon 10 encodes a protein of 645 amino acids, named NSD3 short (NSD3S), which is identical to NSD3L in the first 619 amino acids [12]. Finally, isoform WHISTLE (WHSC1-like 1 isoform 9 with methyltransferase activity to lysine) is a short alternative splice version of the C- terminal of NSD3L that encodes a protein of 506 amino acids. NSD3L has five PHD (plant-homeodomain)-type zinc fingers motifs, two PWWP domains, and the methyltransferase SET domain. Right next to the SET domain there is a SAC (SET-associated Cys-rich) domain rich in cysteines, followed by a Cys-His-rich domain termed C5HCH motif near the C terminal end of the protein [13,14]. The PWWP domain is a histone methyl-lysine (H3K36) reader, acting as an epigenetic regulator of gene expression [15,16,17], and has been postulated as a site for protein–protein interactions due to the amino acid composition [18]. The PHD domain binds chromatin at histone H3 lysine 4 unmodified or methylated [19]. The SET domain is a region conserved between the SET family of methyltransferases, with specificity for mono- or di-methylation of H3 lysine 36. The SET domain is separated into three smaller segments, the pre-SET, SET and post-SET domain, all of which are needed for catalytic activity [20]. Importantly, the post-SET region is essential for binding to nucleosomes [21]. Finally, the PHD5-C5HCH region of NSD3 recognizes the H3 N-terminal peptide containing unmodified K4 and trimethylated K9, which may recognize different HPTMs than NSD1 and NSD2, and may localize this H3K36 methyltransferases to different genome sites [14]. Because NSD3S includes only the N-terminal region of the full-length protein, it only has the first PWWP domain and lacks the methyltransferase activity [12,13] (Figure 1). The NSD3L amino acid sequence reveals a similarity of 68% with NSD1 and 55% with NSD2, in regions with conserved domains (between residues 703 and 1409), including the SET domain [13].

## 3. NSD3 Alterations in Cancer

**Figure 2:**
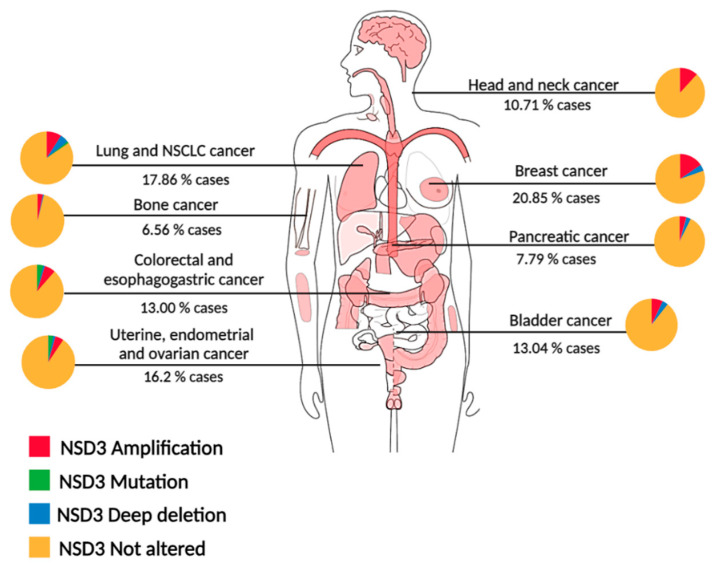
NSD3 genetic alterations across cancer types. Diagram of genetic alterations in pan-cancer analysis of whole genomes (ICGC/TCGA) [26]. Percentages shown under each cancer type indicate the total NSD3 alterations, and each cancer has a pie chart which shows the fraction for each NSD3 alteration, amplifications in red, mutations in green and deep deletions in blue, no alterations in yellow. NSCLC: Non small-cell lung cancer.

NSD3 proteins are ubiquitously expressed in human tissues, with the NSD3S isoform being more prominent than NSD3L [22,23,24], and the WHISTLE isoform primarily found in the testis [25]. Compared to NSD1 and NSD2, NSD3 exhibits a higher genetic variation and amplification in cancer. The oncogenic role of NSD3 is manifested by changes ranging from alterations in expression, such as overexpression and point mutations, as well as fusions with other proteins which result in differences in cellular activity (Figure 2).

### 3.1. Study of the Amplicon 8p11-12: Chromothripsis

Next-generation sequencing and its use in cancer research has eased the identification of a novel type of genomic instability known as chromothripsis. Chromothripsis is a pathological phenomenon by which a series of cluster chromosomal rearrangements occur and are localized in limited regions of the genome in one or several chromosomes. This focal chromosomal scrambling contributes to the initiation of cancer by mediating the overexpression of oncogenes (amplification, translocation, or generation of oncogenic fusions), inactivation of tumor suppressor genes (by loss or disruption), and/or the expression of genes that can contribute to cancer therapy resistance [27,28,29]. Stephens and collaborators found that at least 2–3% of all cancers have chromothripsis [27]. Recently, a study from the Pan-Cancer Analysis of Whole Genomes (PCAWG), the Consortium of the International Cancer Genome Consortium (ICGC), and The Cancer Genome Atlas (TCGA) analyzed patterns of chromothripsis across 38 cancer types using whole-genome sequencing data, and estimated a frequency of 40–60% [30].

The 8p11-12 genomic region spans over 10 megabases (Mb) and encompasses over 50 known genes, including NSD3. Amplification of the 8p11-12 chromosomal region is a common genetic event in many epithelial cancers, thus structural variations, such as chromothripsis of the 8p11-12 genomic region, have clinical and biological implications in multiple malignancies. A study of structural variations in esophageal squamous cell carcinoma (ESCC) found that chromothripsis leads to high-level amplification of FGFR1, LETM2 and NSD3 on chromosome 8. Further functional studies showed that NSD3 knock-down prevented cell proliferation but had no statistical suppression of cell migration and invasion in KYSE150 or TE-1 ESCC cell lines [31]. Together with genetic observations, it was postulated that these genes are amplification targets in ESCC. 

In breast cancer, a study showed earlier undescribed chromothripsis-like patterns spanning the 8p11-12 genomic region and allele-specific DNA amplification events. One of the most common 8p11-12 amplification peaks was in the NSD3 loci, identified using DNA copy number analysis. Dual-color interphase FISH demonstrated extensive intra- and intertumoral heterogeneity in 32 of 47 amplified cases, ranging from neutral DNA copy number (two copies per FISH probe) to high level amplification (up to 50 copies per FISH probe), as well as translocation events with DNA sequences from chromosome 8p on other chromosomes and/or aneuploidy of chromosome 8 [32]. Previously reported amplifications, mutations or fusion proteins involving NSD3 in cancer could be generated by this chromothripsis event. More extensive analysis using whole genome sequencing will contribute to unraveling this phenomenon.

#### 3.1.1. Amplification

Because of the 8p11-12 amplicon found in different epithelial cancers [33], NSD3 has been proposed, among other proteins, as an important oncogene for cancer progression. Using different approaches, such as overexpression of NSD3, small interfering RNA (siRNA) and short hairpin RNA (shRNA)-mediated knockdown against NSD3 in 8p11-12 amplified breast cancer cells, it was found that the loss of NSD3 resulted in a profound loss of the growth and survival of these cells, indicating a function for this protein in regulating survival and transformation [22,34]. In a breast cancer mouse model expressing NSD3 in the mammary epithelium, NSD3 was revealed as a transforming oncogene by exhibiting mammary hyperplasia, dysplasia, and invasiveness [35]. NSD3 has also been proposed as an oncogenic driver in non-small cell lung cancer (NSCLC) [11], lung squamous cell carcinoma (LUSC) [36] and pancreatic ductal adenocarcinoma (PDAC) where the 8p11-12 amplicon has also been found. The studies validated the consistent amplification of NSD3 and showed that the depletion of NSD3 decreases the viability and the colony formation capacity of lung and pancreatic cancer cell lines harboring the 8p amplicon [10,37]. Also, in lung and colon cancer cell lines with NSD3 amplification, the loss of the protein leads to cell apoptosis [38]. Xenograft studies in nude mice implanted with NSD3 knockdown cell lines developed fewer pancreatic and lung tumors [10]. Differential protein expression analysis in LUSC suggested that NSD3 could be a critical driver gene in the recurrent 8p11.23 amplicon that also encompasses the FGFR1 oncogene, due to the unsuccessful response to targeted therapies against FGFR1 [36]. Later, Yuan et al. demonstrated that NSD3 and not FGFR1 is the principal oncogenic driver in LUSC with the 8p11-12 amplification, establishing NSD3 as an important regulator in LUSC tumorigenesis [39]. Altogether, these reports postulate NSD3 amplification as one of the main oncogenic drivers of the 8p11-12 amplicon across cancer types.

#### 3.1.2. Fusion Proteins

Rearrangements involving the short arm of chromosome 8 have been reported and associated with different types of cancer. 

**Figure 3:**
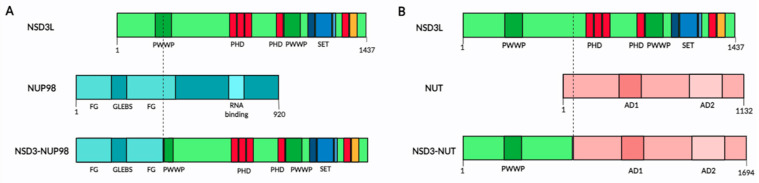
NSD3 fusion proteins in cancer. (**A**) Representation of NSD3-NUP98 fusion protein, indicating in colors the different domains in NSD3 and NUP98 protein. (**B**) Representation of NSD3-NUT fusion protein, indicating in colors the different domains in NSD3 and NUT protein. In NUT protein, AD means acidic domain. In both figures the breakpoint on the proteins is marked as a dotted line. In NSD3L domains, PWWP in green, PHD in red, AWS, SET and post-SET in blue, and C5HCH in orange.

The first NSD3 fusion protein was found in a patient with AML, where the t(8;11) (p11.2;p15) translocation fuses the NUP98 gene to the 3′ end of NSD3 containing both of the PWWP, the SET, PHD and CH5CH rich domains (Figure 3A) [40]. The fusion transcript includes the FG repeats of NUP98, which are known to bind transcription factors, such as CREB-binding protein [40]. This suggests the importance of the transcriptional regulation of leukemic cells and indicates the NUP98-NSD3 fusion into a vital leukemogenesis-related oncogene. The presence of the NUP98-NSD3 fusion protein has been observed in leukemia cell lines and has also been found in B-lymphocyte cell lines derived from healthy volunteers who had undergone transformation by the Epstein–Barr virus [41].

In pediatric sarcoma, investigators found a novel NSD3-NCOA2 fusion. These two proteins have been found to be involved in fusion processes, NSD3 in acute myeloid leukemia and NCOA2 in infantile spindle cell rhabdomyosarcoma, which strengthens the findings and leaves the characterization of its function as well as the presence in other human samples pending [42].

The nuclear protein of the testis (NUT) midline carcinoma (NMC) is an epithelial cancer that is defined by chromosomal translocations of the NUT gene. In about 65% of cases, NUT is fused to BRD4, with 25% fused to BRD3, and the rest 10% unknown, with recent reports showing it to be fused to NSD3 [43,44,45,46]. The first NMC patient with a NSD3-NUT fusion t(8;15)(p12;q15) was identified in 2014. The fusion resulted in a protein containing exons 1–7 of NSD3 and exons 2–7 of NUT, encoding 1694 amino acids, containing amino acids 1–569 of NSD3 and 8–1132 of NUT (Figure 3B) [43]. Unlike the NUP98-NSD3 fusion, the NSD3-NUT fusion has only the N-terminal region of NSD3 protein (the complete NSD3S isoform) without methyltransferase activity. The NSD3-NUT oncofusion is necessary and sufficient for the blockage of differentiation and for the proliferation of NMC cells [43]. Additionally, the same NSD3-NUT oncofusion has been described in patients with NMC of the lung [44].

#### 3.1.3. Mutations

Xiong et al. described a variety of NDS3 missense mutations and T419Pfs*8/Nfs*28/N mutations in four cases of stomach adenocarcinoma (STAD), two cases of colon adenocarcinoma (COAD) and single cases of breast invasive carcinoma (BRCA) and pancreatic adenocarcinoma (PAAD). Likewise, nonsense mutations in NSD3, such as, E1181K and T2342A, enhance the growth of cancer cells and xenograft tumors by disrupting an autoinhibitory loop in the NSD3 protein, thereby increasing enzymatic activity [47,48]. More studies regarding the mutations found in NSD3 are necessary to identify the significance of these mutations on cancer progression.

## 4. NSD3 Involvement in Cancer

**Figure 4:**
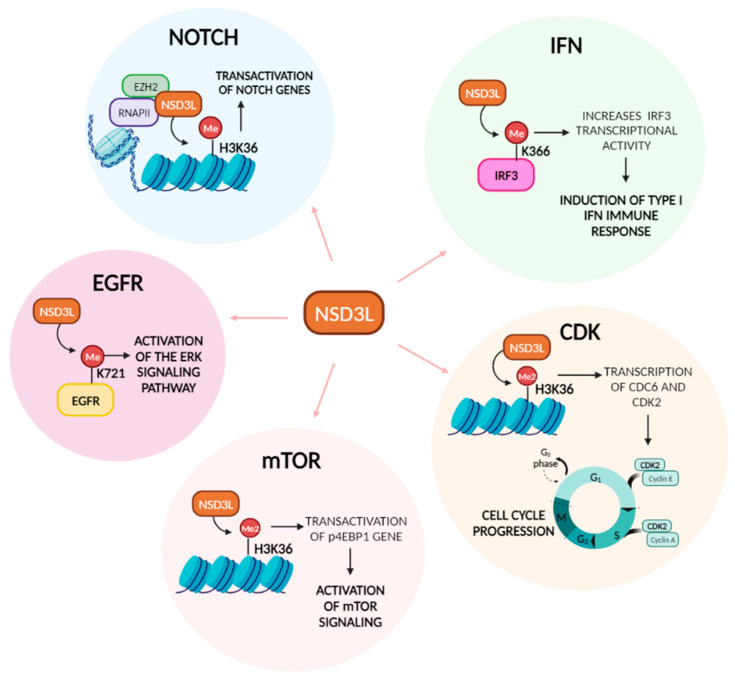
NSD3L functions in cancer pathways. Diagram showing the methyltransferase-dependent functions of NSD3L in the different cancer pathways, specifically indicating where NSD3L acts on the pathway and the outcome.

It is well known that NSD3 catalyzes the methylation of histone H3 at lysine 36, this occurs because NSD3 binds to LSD2 and G9a/EHMT2, forming a complex in vivo [49]. G9a and LSD2 mediate H3K9 methylation and H3K4 demethylation of actively transcribed genes, helping NSD3 to recognize and methylate H3K36 [14]. Morishita et al. used the C-terminal portion of NSD3, including pre-SET, SET, post-SET and PHD5 domain and identified that in vitro, NSD3 can methylate H3K9, H3K27, H3K36, H3K79 and H4K20 [4]. Discrepancies about the specificity for the substrate of the catalytic domain of NSD3 and other members of the NSD family, may be due to the cellular context, the assay employed, the nature of the substrate, or the portion of the protein used, if it is only the SET domain or the full-length protein. In relation to the isoforms of NSD3, NSD3L has been associated with neural crest formation and migration, playing a role in gene expression during neural crest development [50,51]. NSD3S conserves the N-terminal PWWP domain, this domain allows the protein to bind histone H3 at methylated lysine 36 [52]. The WHISTLE isoform has been found to act as a transcriptional repressor through HDAC1 recruitment, having H3K4me2 and H3K27me2/3 methyltransferase activity [25,53], this isoform is considered less relevant to cancer. To better understand how the two main isoforms of NSD3 carry out their oncogenic role, we classified the different functions of NSD3 as methyltransferase-dependent (NSD3L) (Figure 4) or as an adaptor protein function (NSD3S).

### 4.1. Methyltransferase-Dependent Function of NSD3 in Cancer

#### 4.1.1. NOTCH Pathway

NSD3L interacts with EZH2 and RNA polymerase II to influence H3K36me2/3-dependent transactivation of genes, including those related to NOTCH signaling in breast cancer with the 8p11-12 amplicon, such as NOTCH receptors, ligands, and ADAM12 [54]. These findings indicate that NSD3-induced methylation of H3K36 activates NOTCH signaling to drive breast tumor initiation and metastatic progression.

#### 4.1.2. mTOR Pathway

Deregulation of the mTOR pathway occurs in various diseases, including cancer. The mTOR pathway responds to environmental signals, regulating basic cell functions like cell growth and proliferation [55], survival, apoptosis, angiogenesis, and metabolism [56]. Yuan et al. showed that in lung cancer, with the amplification of chromosomal region 8p11-12, methylation of H3K36 by NSD3 resulted in the transcription of key oncogenic genes, including those involved in mTOR signaling activation [39]. In their studies, in mice tumors driven by the mNSD3T1242A mutation, RNAseq analysis showed upregulation of MYC, BRD4, and p4EBP1, the last one being involved in activation of mTOR signaling. In pancreatic cancer, NSD3 silencing resulted in inhibition of S6K1 phosphorylation, indicating that in the absence of the NSD3 oncoprotein mTOR was not activated [48].

#### 4.1.3. EGFR Pathway

It has been shown that HMTs methylate not only histones, but also proteins. The NSD family of proteins have also been described as performing that function, as both NSD1 [57] and NSD2 [58] methylate NF-kB to regulate its function.

NSD3-mediated mono-methylation of the EGFR kinase domain (Lys721) affects the cytoplasmic and nuclear function of the protein. In the cytoplasm, it increases EGFR kinase activity and the downstream ERK signaling pathway without the presence of the ligand EGF. In the nucleus, it stimulates cell cycle progression by increasing the binding of EGFR to PCNA on squamous cell carcinoma of the head and neck (SCCHN) cancer cells [59]. A study in colorectal cancer (CRC) cells showed that overexpression of NSD3 increased phosphorylation of ERK, leading to enhanced proliferation and migration of CRC cells [60]. Xiong et al., in pancreatic cancer cells, also found a correlation between decreased EGFR/ERK pathway activation and NSD3 knockdown [47].

#### 4.1.4. IFN Pathway

Activated Interferon regulatory factor 3 (IRF3) is a transcriptional regulator that promotes IFN-α and IFN-β transcription. IFN-β elicits both anti-inflammatory and pro-inflammatory responses, playing a key role in innate immunity and the response to viral infections [61,62]. Importantly, NSD3-deficient mice are more susceptible to viral infection. Primary peritoneal macrophages derived from NSD3-deficient mice have lower IFN-β, IL-6, IL-8, and TNF levels upon VSV infection [63]. NSD3 was found to interact with IRF3 using its PWWP domain, and methylates IRF3 at K366 in VSV-infected HEK293T cells. NSD3-mediated methylation increases IRF3 transcriptional activity by interfering with binding between IRF3 and the PP1cc phosphatase, thereby maintaining IRF3 phosphorylation and activity. Therefore, NSD3 acts as a critical promoter for the induction of type I IFNs and antiviral innate immune response [63].

It is accepted that innate and adaptive immunity plays an important role in antitumor immune surveillance. Amplification of NSD3 in patients with LUSC exhibits a decrease in the type II IFN response, leading to an immune–desert pro-tumorigenic phenotype [64]. In breast cancer, increased expression of NSD3 correlated with a decrease CD8+ T cells and increased PD-L1 gene expression [65], in agreement with findings from Xu et al. in lung cancer. In contrast, bioinformatic studies in PAAD have shown that upregulation of NSD3 correlated with increased immune infiltration, specifically macrophages, B cells, neutrophils, CD8+ T cells and dendritic cells [47]. Taken together, there is evidence linking the role of NSD3 in IFN response and immune modulation, however, further studies are needed to accurately state the function.

#### 4.1.5. Cyclin Dependent Kinase (CDK) Pathway

CDC6 and CDK2 promote G1 to S phase transitions, and the transcription of these genes is regulated by H3K36 di-methylation. In SCCHN cells, it was demonstrated that NSD3 regulates transcription of CDC6 and CDK2, as knockdown of NSD3 resulted in G0/G1 arrest [66]. Knockdown of NSD3 by siRNA in bladder and lung cancer cell lines reduced cell proliferation by inducing cell cycle arrest at G2/M phases through the regulation of the expression of CCNG1 and NEK7, which are important regulators of G2/M transition in cancer cells [67,68,69]. In a human osteosarcoma cell line, NSD3 silencing resulted in the inhibition of cell proliferation, induction of apoptosis, and an increase in the number of cells in th eG2/M phase, suggesting a role for NSD3 in G2/M cell cycle arrest [70]. NSD3L depletion by siRNA resulted in an increased number of cells with separated sister chromatids during prometaphase, caused by spindle assembly checkpoint dependent arrest. Also, depletion of NSD3L resulted in defective sister chromatid cohesion in G2 cells, implying that the NSD3L isoform acts in a cell cycle checkpoint before mitosis by decreasing cohesin and MAU2 recruitment onto chromatin [71].

### 4.2. NSD3S Isoform Function as an Adaptor Protein

#### 4.2.1. NSD3-NUT Fusion

The NSD3-NUT fusion oncoprotein is present in several NMC cases. After knockdown of endogenous NSD3-NUT in an NMC cell line, there was an increase in keratin levels, and a decrease in cellular proliferation, indicating a crucial role of the NSD3-NUT oncofusion in blocking cell differentiation and stimulating the proliferation in this cell line. It was also found that NSD3 not only interacts with NUT, but is associated with BRD4-NUT fusion, this interaction being important in the blockade of differentiation [43]. It is known that BRD4-NUT binds and activates the histone acetyltransferase, p300, leading to the inactivation of p53 [72]. Recently, the predominance of the NSD3-NUTM1 oncofusion in thyroid NUT carcinoma was described [73,74].

#### 4.2.2. NSD3S-BRD4-CHD8 Interactions

NSD3 imparts a pTEFb-independent transcriptional activation function on BRD4, on genes such as CCND1 and PIM2. The BRD4/NSD3 complex regulates the methylation of H3K36 at BRD4 target genes [75]. BRD4 regulates the transcription of some genes, like CD274 that encodes PD-L1 [76]. NSD3S was described as an adaptor protein by Shen et al. that characterized the binding of BRD4 to a small 11 amino acid region on the N-terminal of NSD3 (amino acid 152–163). Also, they showed that the short isoform, NSD3S, was required and sufficient for driving leukemia progression, indicating a methyltransferase independent function of the protein. NSD3S also binds to the chromatin remodeler CHD8 through the C-terminal region, linking BRD4 to CHD8 on the chromatin, through the ET domain of BRD4 [23]. The three proteins colocalize in regions of the genome and they are release from MYC super-enhancers using BET inhibitor, JQ-1 [23].

#### 4.2.3. NSD3S-MYC Interaction

**Figure 5:**
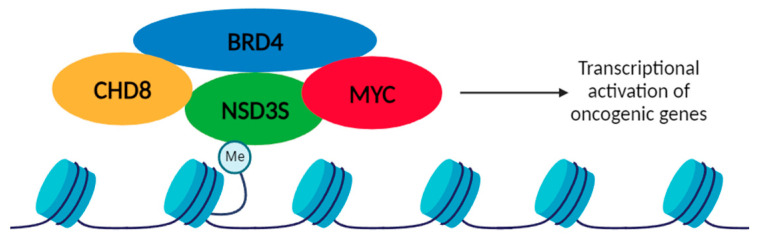
Proposed model for the oncogenic role of NSD3S as an adaptor protein. NSD3S binds to methylated histones (H3K36) through the PWWP domain and could recruit oncogenic proteins, such as CHD8, BRD4 and MYC, activating an oncogenic transcriptional program. NSD3S adaptor function is shown as interacting with MYC, BRD4 and CHD8.

Sun et al. reported that in NSD3 knockout pancreatic cells and in shRNA-xenografts, there was a decrease in the gene expression of *Myc*, *Adam12*, and *Notch3*, demonstrating that the silencing of NSD3 can downregulate oncogenic genes [48]. We described that NSD3S interacts with MYC to stabilize the MYC protein and increase its transcriptional activity, acting as an oncogenic interaction [77]. The NSD3S-MYC interaction is mediated by a 15 amino acid site on NSD3S, between amino acids 389 and 404. Interestingly, deletion of the 15 amino acid region showed decreased stabilization of MYC, through the suppression of the FBXW7 mediated the degradation of MYC [78]. It is well established that BRD4 regulates transcription of MYC in cancer [79]. Moreover, MYC interacts with BRD4 through the internal region of the protein and catalyzes the phosphorylation of Thr58, resulting in MYC ubiquitination and degradation [76,80]. The connection between NSD3S-MYC-BRD4, and potentially other oncogenic signaling proteins, must still be deciphered, but it can be postulated that NSD3S may act as a scaffolding protein that recruits oncogenes to chromatin by binding to H3K36 through the PWWP domain (Figure 5).

## 5. Discussion

NSD3 is present as three isoforms, with the NSD3L and NSD3S proteins being linked to cancer. Importantly, NSD3L has catalytic activity, whereas NSD3S only has the first PWWP domain that binds to H3K36 marks on the chromatin. During the last few years, NSD3 has been extensively studied in relation to cancer; however, knowledge about the specific roles of the various isoforms in cancer progression remains unknown, and the currently available information focuses on NSD3 function depending on the tumor being studied. Moreover, as both isoforms are ubiquitously expressed in human tissues, there is to date no evidence showing that one isoform may play a more significant role over the other.

Chromothripsis events have been reported since 2012, causing multiple alterations that result in gene amplification, deletion, mutation, or fusion. Moreover, in cancer the correlation between chromothripsis and NSD3-amplification (8p11-12 amplicon) was established in 2018. NSD3 has been postulated as one of the main oncogenic drivers of the amplicon 8p11-12 across cancer types. Interestingly, it has been reported that about 50% of invasive breast tumors that have the 8p11-p12 amplicon also harbor MYC amplification [81]. This suggests that patients with 8p11-12 and 8q24.21 co-amplification may present with a more aggressive phenotype. More research is needed to see the prevalence between these two types of alteration across cancer types that could help the prognosis of patients.

As previously mentioned, NSD3 has been well established as an oncogene; however, there are still not enough studies that differentiate the isoform-specific contributions of NSD3 in cancer progression. The NSD3L isoform contains the catalytic domain, mediating the methylation of H3K36 and activating gene transcription. In relation to cancer, NSD3L can also methylate proteins. Specifically, it has been reported that the methylation and activation of EGFR and IRF3 promotes an oncogenic function of these proteins. There are reports of other NSD members methylating and activating oncogenic proteins, such as p65 of the NF-kB pathway [57] and STAT3 [82], suggesting the possibility that NSD3L could methylate and regulate other proteins. We believe that the methylation of non-histone proteins mediated by the NSD family should be further studied in the context of cancer.

Over the past several years, NSD3S has been established as an adaptor protein for important drivers of cancer, such as MYC, BRD4, and CHD8. NSD3S binds to chromatin through the PWWP domain and could recruit MYC, along with other chromatin regulators, to increase transcription of MYC target genes, possibly inducing an oncogenic phenotype. Moreover, NSD3S has been shown to be sufficient for leukemia progression, it also stabilizes and increases MYC transcriptional activity and is present on the oncogenic NUT fusion. There are some preliminary reports on NSD3S involvement in the Wnt pathway [22,83]; however, further studies should be taken into consideration. According to the current findings, we conclude that histone or non-histone protein methylation may be one of the oncogenic mechanisms of NSD3, although not the only one. Certainly, there are functions of NSD3 independent of the SET domain.

Due to the oncogenic activity of NSD3 isoforms, driven through the methyltransferase activity or acting as an adaptor protein, NSD3 has been studied as a therapeutic target in cancer. Inhibitors against NSD3 have been developed since 2019, specifically targeting the PWWP1 domain of NSD3 (BI-9321), inhibiting both isoforms of the protein. Interestingly, BI-9321 decreases the expression of the *myc* gene and reduces cell proliferation [84]. Later, two studies reported the discovery and characterization of NSD3 PROTAC, being more efficient in blocking NSD3 function and decreasing MYC oncogenic node. Both PROTAC were synthesized incorporating an NSD3-PWWP antagonist link to an E3 ligase, showing the degradation of both NSD3 isoforms in cell lines of AML, multiple myeloma, and lung cancer [85,86]. These studies correlate with our findings that NSD3S/MYC interaction stabilizes and activates MYC transcriptional activity. Finally, Kim et al. reported the identification of a new NSD3 inhibitor which targets the SET domain of the protein, therefore only the NSD3L isoform [87]. However, based on the understanding we have regarding the function of NSD3, particularly NSD3S, we believe that inhibitors targeting both isoforms would be more effective in blocking oncogenic programs in a therapeutic context. Despite the absence of clinical studies utilizing NSD3 inhibitors, it remains compelling to investigate the clinical outcomes of, particularly, NSD3S inhibitors in NSD3 dysregulated cancers. Our proposed model highlights the interaction between the adaptor protein NSD3S and oncogenic drivers such as MYC, BRD4, and CHD8. In recent years, the development of inhibitors targeting the bromodomain and extraterminal proteins (BET), such as BRD4, is exponentially expanding [88]. A recent phase-2 clinical trial regarding the safety and effectiveness of a BET inhibitor (ZEN-3694) in squamous cell lung cancer patients with NSD3 amplification is currently ongoing (http://www.clinicaltrials.gov (accessed on 2 January 2024), identifier NCT05607108). They propose that BET inhibition may counteract the effect of NSD3 on tumor growth. The pharmacological disruption of the BRD4-NSD3S complex is a novel therapeutic target that is currently underway, with further potential applications in various cancer types that rely on NSD3 activity.

Given the importance and the prevalence of NSD3 amplifications across several types of cancer, a combination of whole genome sequencing, isoform specific pharmacological targeting, and the study of the interactome of NSD3 will help to understand the altered expression and contribution of both isoforms of NSD3 in cancer.

## Data Availability

Not applicable.
